# Gene length corrected trimmed mean of M-values (GeTMM) processing of RNA-seq data performs similarly in intersample analyses while improving intrasample comparisons

**DOI:** 10.1186/s12859-018-2246-7

**Published:** 2018-06-22

**Authors:** Marcel Smid, Robert R. J. Coebergh van den Braak, Harmen J. G. van de Werken, Job van Riet, Anne van Galen, Vanja de Weerd, Michelle van der Vlugt-Daane, Sandra I. Bril, Zarina S. Lalmahomed, Wigard P. Kloosterman, Saskia M. Wilting, John A. Foekens, Jan N. M. IJzermans, on behalf of the MATCH study group, Peter Paul L. O. Coene, Jan Willem T. Dekker, David D. E. Zimmerman, Geert W. M. Tetteroo, Wouter J. Vles, Wietske W. Vrijland, Rolf Torenbeek, Mike Kliffen, J. H. Carel Meijer, Anneke A. vd Wurff, John W. M. Martens, Anieta M. Sieuwerts

**Affiliations:** 1 Department of Medical Oncology, Erasmus MC Cancer Institute, Erasmus MC University Medical Center, 3015 CE Rotterdam, The Netherlands; 2 Department of Surgery, Erasmus MC University Medical Center, 3015 CE Rotterdam, The Netherlands; 3 Cancer Computational Biology Center, Erasmus MC Cancer Institute, Erasmus MC University Medical Center, 3015 CE Rotterdam, The Netherlands; 4 Department of Urology, Erasmus MC Cancer Institute, Erasmus MC University Medical Center, 3015 CE Rotterdam, The Netherlands; 5 Department of Genetics, Center for Molecular Medicine, University Medical Center Utrecht, 3584 CX Utrecht, The Netherlands; 6 Cancer Genomics Center, 3584 CG Utrecht, The Netherlands

**Keywords:** RNA sequencing, Normalization methods, GeTMM, edgeR, TPM, DESeq2, Colorectal Cancer

## Abstract

**Background:**

Current normalization methods for RNA-sequencing data allow either for intersample comparison to identify differentially expressed (DE) genes or for intrasample comparison for the discovery and validation of gene signatures. Most studies on optimization of normalization methods typically use simulated data to validate methodologies. We describe a new method, GeTMM, which allows for both inter- and intrasample analyses with the same normalized data set. We used actual (i.e. not simulated) RNA-seq data from 263 colon cancers (no biological replicates) and used the same read count data to compare GeTMM with the most commonly used normalization methods (i.e. TMM (used by edgeR), RLE (used by DESeq2) and TPM) with respect to distributions, effect of RNA quality, subtype-classification, recurrence score, recall of DE genes and correlation to RT-qPCR data.

**Results:**

We observed a clear benefit for GeTMM and TPM with regard to intrasample comparison while GeTMM performed similar to TMM and RLE normalized data in intersample comparisons. Regarding DE genes, recall was found comparable among the normalization methods, while GeTMM showed the lowest number of false-positive DE genes. Remarkably, we observed limited detrimental effects in samples with low RNA quality.

**Conclusions:**

We show that GeTMM outperforms established methods with regard to intrasample comparison while performing equivalent with regard to intersample normalization using the same normalized data. These combined properties enhance the general usefulness of RNA-seq but also the comparability to the many array-based gene expression data in the public domain.

**Electronic supplementary material:**

The online version of this article (10.1186/s12859-018-2246-7) contains supplementary material, which is available to authorized users.

## Background

In recent years, the analysis of the transcriptome has switched from using microarrays to the potentially more powerful and informative massive parallel sequencing of cDNA (RNA-seq) [1]. In RNA-seq, sequence reads are aligned to a reference genome, and the number of reads mapping to a feature – such as a gene – is a measure which is proportional to both the length and abundance of said feature. Before performing downstream analyses, normalization has to be performed to correct for differences between sequencing runs (e.g. library size and relative abundances).

Current normalization methods allow for either inter- or intrasample comparison. The two most commonly used normalization methods when interested in DE genes between samples (intersample comparison) are edgeR [2] and DESeq [3, 4]. The normalization algorithms of these 2 methods (Trimmed Mean of M-values, TMM, for edgeR and Relative Log Expression, RLE, for DESeq) show consistent good performance compared to other normalization algorithms (Total count, UpperQuartile, Median, Quantile, and those employed by LimmaQN, limmaVoom, CuffDiff and Bayseq) [5–8]. Notably, TMM and RLE do not correct the observed read counts for the gene length, which is theoretically irrelevant for intersample comparisons. However, this approach does not allow for intrasample comparison, because a longer gene will get more read counts compared to a shorter gene when expressed at equal levels. Thus, samples can seem highly correlated without correction when in fact the correlation is much lower after length correction (see Additional file 1), and in extremis can be correlated based on gene length instead of the expression levels. This problem extends to correlation based methods where for example a panel of genes of a sample is correlated to another sample, as is often done in hierarchical clustering (correlation is used as similarity metric). Furthermore, classifiers based on correlation of an established signature gene panel to a new sample such as the consensus molecular subtypes (CMS) in colorectal cancer will yield erroneous results without correcting gene expression levels for gene length.

The most commonly used normalization method that includes gene length correction is TPM (Transcripts Per kilobase Million) [9], as other methods like RPKM [1]/FPKM [10] (Reads/Fragments Per Kilobase per Million reads, respectively, proved to be inadequate and biased [5, 6, 11, 12].

Ideally, a normalization method should generate a data set on which both between-sample and within-sample analyses can be performed. We therefore introduce GeTMM (Gene length corrected TMM), a novel normalization method combining gene-length correction with the normalization procedure TMM, as implemented in edgeR, to allow both inter- and intrasample comparison with the same normalized data set. We used true (i.e. not simulated) RNA-seq data of a large cohort of primary tumors of 263 colon cancer patients, and normalized these data using our new method GeTMM, alongside TMM, RLE and TPM [6]. We investigated several properties of the normalized data sets with regard to distribution, effect of RNA quality, subtype-classification (i.e. the CMS classification) [13], a clinical recurrence score [14], recall of DE genes and correlation to RT-qPCR data generated from the same samples. The main objective of this study was to determine if GeTMM performs equivalent to the other normalization methods with regard to intersample analyses, and if and to what extent gene length correction influences intrasample analyses.

## Methods

### Description of cohort

Fresh-frozen tumor tissue of 263 colon cancer patients of the MATCH study, a multicenter observational cohort study, who underwent surgery in one of seven hospitals in the Rotterdam region, the Netherlands, were used. Inclusion criteria and additional clinical characteristics have been described [15].

### RNA isolation, cDNA synthesis, qPCR and RNA-seq

Detailed description of the RNA-isolation has been described previously [16, 17]; briefly, RNA was isolated from 30 μm sections using RNA-Bee® according to the manufacturer’s instructions (Tel-Test Inc., USA). Quality and quantity of RNA before and after genomic DNA (gDNA) removal and clean-up with the NucleoSpin RNA II tissue kit (Macherey-Nagel GmbH & Co. KG, Germany) were assessed with the Nanodrop ND-1000 (Thermo Scientific, Wilmington, USA) and the MultiNA Microchip Electrophoresis system (Shimadzu, Kyoto, Japan). RNA Integrity Numbers (RIN) were assessed using the MultiNA Microchip Electrophoresis system after gDNA removal and clean-up (Additional file 2 evaluates the relation between Agilent’s BioAnalyzer RIN value and the quality as measured by MultiNA). cDNA was generated from 1 μg total RNA with the RevertAid H Minus First Strand cDNA synthesis kit according to the manufacturer’s instructions (Fermentas, St Leon-Rot, Germany). RT-qPCR was performed with the Mx3000P QPCR machine (Agilent Technologies, the Netherlands) using ABgene Absolute Universal or Absolute SYBR Green with ROX PCR reaction mixtures (Thermo Scientific, USA) according to the manufacturer’s instructions. The intron-spanning assays to quantify levels of 33 transcripts by the delta-delta Cq method were assessed as described before [16, 17] and are summarized in Additional file 3.

For RNA-seq, 500 ng of total RNA after gDNA removal, clean-up and removing ribosomal RNA using Ribo Zero (Illumina, USA), was used as input for the Illumina TruSeq stranded RNA-seq protocol (paired-end). No biological replicates were used. Libraries were pooled and sequenced on Illumina HiSeq2500 (2x101bp, 177 samples) or NextSeq (2x76bp, 86 samples) instruments. Pool sizes and the amount of samples per run were determined based on the percentage of tumor cells estimated from histological examination [15]. We used the STAR [18] algorithm (version 2.4.2a) to align the RNA-seq data on the GRCh38 reference genome (settings are in Additional file 4). To obtain read counts for each gene, the ‘quantMode GeneCounts’ was used, in which only those reads that have a sufficient alignment score and those that are uniquely mapped are included. The 76 bp read length from the NextSeq machine was more than sufficient for accurate mapping to the reference genome, and we found no bias in data originating from the different machines.

Gene annotation was derived from GENCODE Release 23 (https://www.gencodegenes.org/). To obtain exon specific counts for *CDK1* and *MKI67*, all unique HAVANA exons for each gene were extracted and used in FeatureCounts [19] with the following settings “–t exon”, -O and –f. These settings, and the absence of –p (for paired-end counting), ensures that reads that overlap multiple exons are counted for each of these exons. This ensured all evidence for the presence of an exon was counted.

### Normalization of RNA-seq data

The raw read counts of all samples were merged in a single read count matrix. This matrix was used as input for each of the different normalization methods. The most commonly used RNA-seq normalization methods are TMM, implemented in edgeR [2] and RLE, in DESeq2 [3, 4]. Both these methods do not employ any gene length normalization since their aim is to identify DE genes between samples and thus assume that the gene length is constant across samples. The TPM method adds to the previously used RPKM - for single-end sequencing protocols - or its paired-end counterpart FPKM. TPM uses a simple normalization scheme, where the raw read counts of each gene are divided by its length in kb (Reads per Kilobase, RPK), and the total sum of RPK is considered the library size of that sample. Next, the library size is divided by a million, and that is used as scaling factor to scale each genes’ RPK value. Thus, TPM does correct for gene length, but is lacking a sophisticated between-sample correction; it does not account for a possible small number of highly expressed genes, thus comprising a large portion of the total library size of that sample. DESeq2 and edgeR address this problem by estimating correction factors that are used to rescale the counts (see [2, 3] for more details). In short, edgeR employs the Trimmed Means of M values (TMM) [2] in which highly expressed genes and those that have a large variation of expression are excluded, whereupon a weighted average of the subset of genes is used to calculate a normalization factor. DESeq2 uses RLE that also assumes most genes are not DE; here, for each gene the ratio of its read count in a sample over the geometric mean of that gene in all samples is calculated. The median of the ratios of all genes in a sample is used as correction factor. Where TMM (edgeR) estimates a correction factor that is applied to the library size, the correction factor of RLE (DESeq2) is applied to the read counts of the individual genes.

**Fig. 1:**
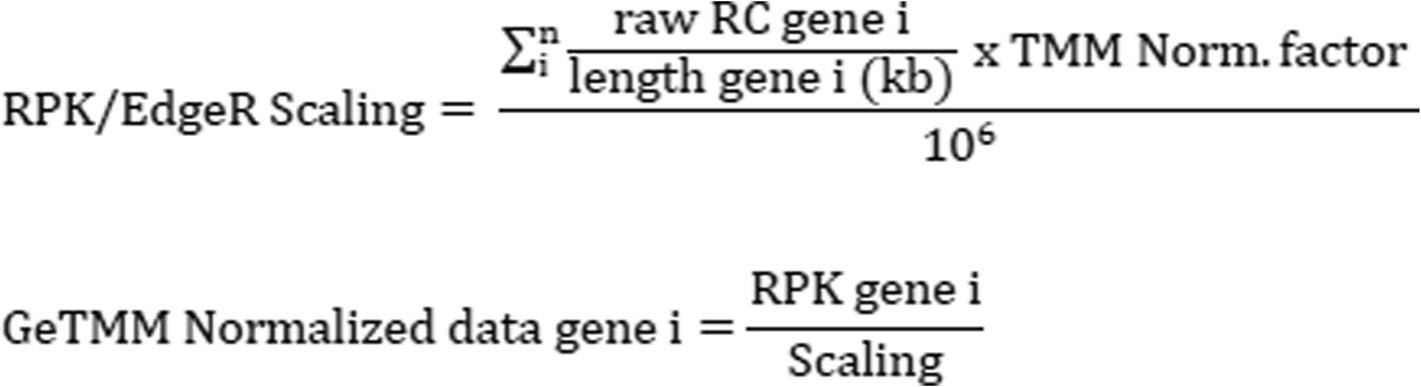
normalization using GeTMM method with *n =* number of genes and i = given gene i

Such normalized data are better comparable between samples, but still suffer from the inability to compare gene expression levels within a sample. To obtain a normalized data set that is equally suitable for between-samples and within-sample analyses, the following GeTMM method is proposed: first, the RPK is calculated for each gene in a sample: raw read counts/length gene (kb). In edgeR, which uses TMM-normalization, normally the library size (total read count; RC) is corrected by the estimated normalization factor and scaled to per million reads, but in GeTMM the total RC is substituted with the total RPK (Fig. 1).

In practice, to obtain GeTMM normalized data, pre-calculate the RPK values from the raw read counts and gene length (in kb), and use these values as input for the edgeR package. See Additional file 4 for a step by step procedure in R. The gene length is calculated using the annotation by gencode: the length of all exons with a unique exon_id annotated to the same gene_id is summed. DESeq2 only allows integers as input, thus the fractions generated by the gene length correction are rejected for input by DESeq2.

edgeR and DESeq2 are available as R-packages (https://bioconductor.org/), and subsequent analyses were performed using R (v3.2.2). To obtain normalized data, the raw read count matrix (tab-delimited text file) was used as input. R commands to obtain normalized data are listed in Additional file 4. Each method outputs normalized read counts, that were log2-transformed (setting genes to NA when having 0 read counts).

The CMS classification was performed using the “CMSclassifier” package (https://github.com/Sage-Bionetworks/CMSclassifier), using the single-sample prediction parameter. The Onco*type* DX® [14] recurrence score was performed as described for the RT-qPCR data, and using the RNA-seq normalized values as input for the algorithm. In short, expression data of 7 genes are used; *BGN, FAP, INHBA* (stromal panel), *MKI67, MYC, MYBL2* (cell cycle panel) and *GADD45B.* An unscaled recurrence score (Rsu) is calculated as (0.1263 x average stromal panel) – (0.3158 x average cell cycle panel) + (0.3406 x *GADD45B)*. The Recurrence Score (RS) is calculated as 44.16 x (Rsu + 0.30). The signal-to-noise ratio (SNR) was calculated as the (mean1 – mean2)/Sp, where Sp is the square root of the pooled variance Vp. This is calculated as Vp = [(n1–1) V1 + (n2–1)V2]/(n1 + n2–2), where V1 and V2 are the variance for each of the groups, and n1 and n2 the sample group sizes.

### Statistics

Statistical tests were performed using R (v3.2.2), using non-parametric tests (Mann-Whitney U test, Spearman rank correlation) where appropriate. For identifying DE genes, the default tests that are included within the edgeR and DESeq2 packages were used (a Wald test for DESeq2 and for edgeR an exact test for the negative binomial distribution). For edgeR, a common dispersion value of 0.4 was used, as suggested by the documentation. Additionally for edgeR and DESeq2, but also for RT-qPCR, TPM and GeTMM the Student’s t-test was used. For the calculation of Root Mean Square Error (RMSE), standardized data were used (Z-normalization, subtracting the mean expression value of a gene from the observed expression value in a sample, and dividing this by the standard deviation of the gene’s expression values). Statistical tests are indicated in the main text, *p*-values were two-sided and p-values and FDRs (Benjamini-Hochberg, when required) were considered significant when below 0.05.

## Results

We used primary tumor tissue of a cohort of 263 colon cancer patients to generate RNA-seq data. There were no biological or technical replicates. We aligned these data to the human reference genome (GRCh38) and generated read counts per gene. This read count matrix was used for several normalization procedures: TMM (implemented by edgeR) [2], RLE (implemented by DESeq version 2) [3] and TPM, in addition to a newly proposed method of gene length correction in combination with the normalization used by edgeR - GeTMM. To validate the results, the same RNA used for generating the sequence libraries was also used for RT-qPCR analysis of 33 genes (see Additional file 3 for details). Our study was not designed to identify the method with the highest compatibility to RT-qPCR data, but to compare the performance of GeTMM to the other normalization methods in inter- and intrasample analyses.

### Distribution of RNA-seq data

**Fig. 2:**
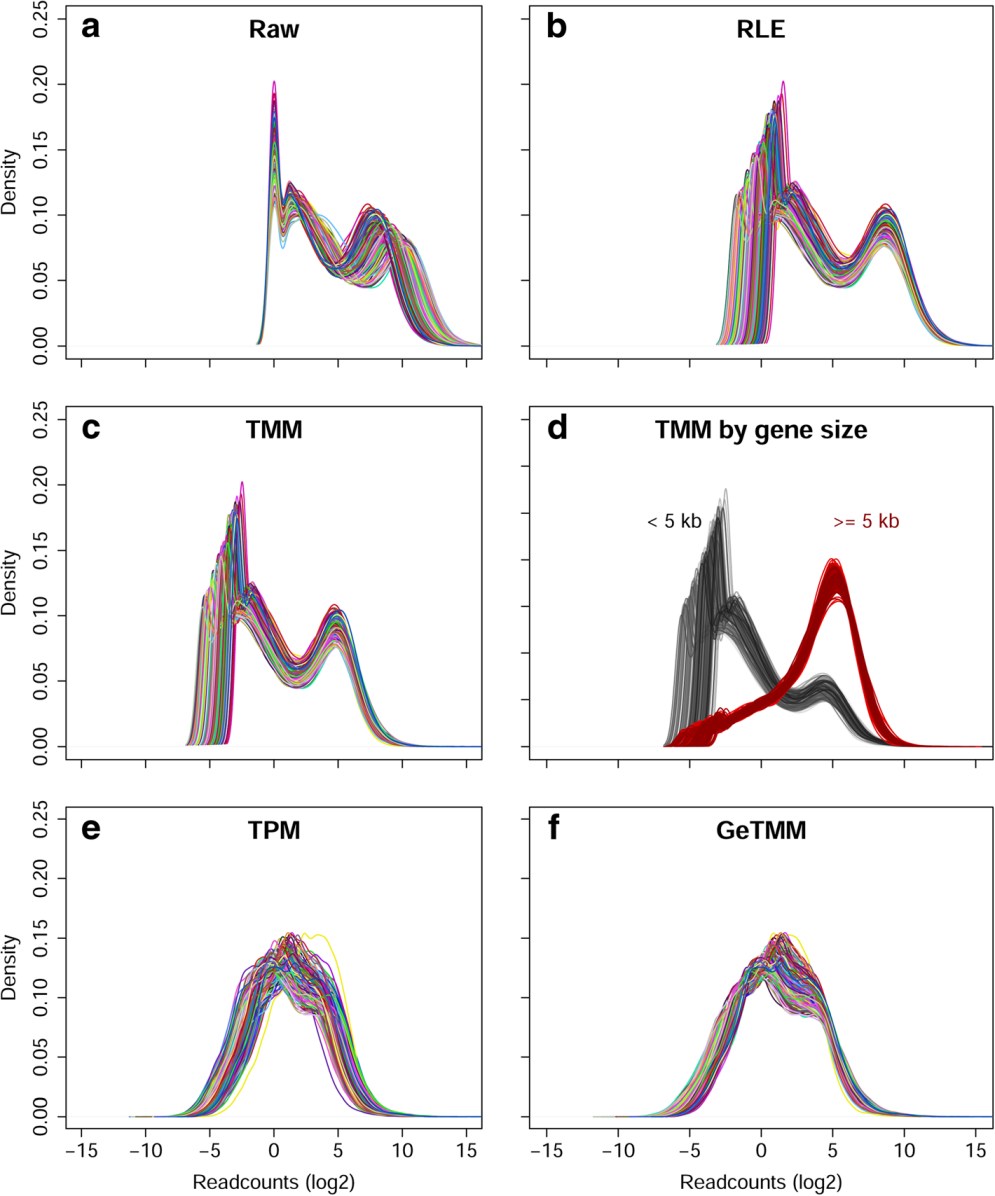
Density plot by normalization method. Each line corresponds to the distribution of expression levels in a sample. X-axis shows log2 of read counts. **a**-**f** respectively show the distribution without normalization, and normalization according to several methods, as indicated

The library sizes (i.e. the number of mapped reads) of the samples ranged from 5.8 to 37.8 million (mean 16.0 million and median 14.2 million). Density plots were generated to get an overview of the read count distributions (Fig. 2). Panel 2a shows the raw read counts (not normalized, in log2 scale), which clearly shows a bimodal distribution after the initial peak at 0, with peaks at 1.1~ 1.4 log2-read counts and a broader peak at 7~ 10 log2-read counts. Similar bimodal distributions were seen after RLE and TMM normalization, respectively by DESeq2 and edgeR (Fig. 2b, c), which both do not correct for gene length. Splitting the TMM normalized data by genes < 5 kb and those > = 5 kb (Fig. 2d) shows that the bimodality is largely attributable to the gene length; as expected, longer genes generally have higher read counts. Methods employing correction for gene length - TPM and GeTMM - both show a more Gaussian distribution (Fig. 2e, f).

### Comparison to RT-qPCR generated data: Intersample analysis

**Fig. 3:**
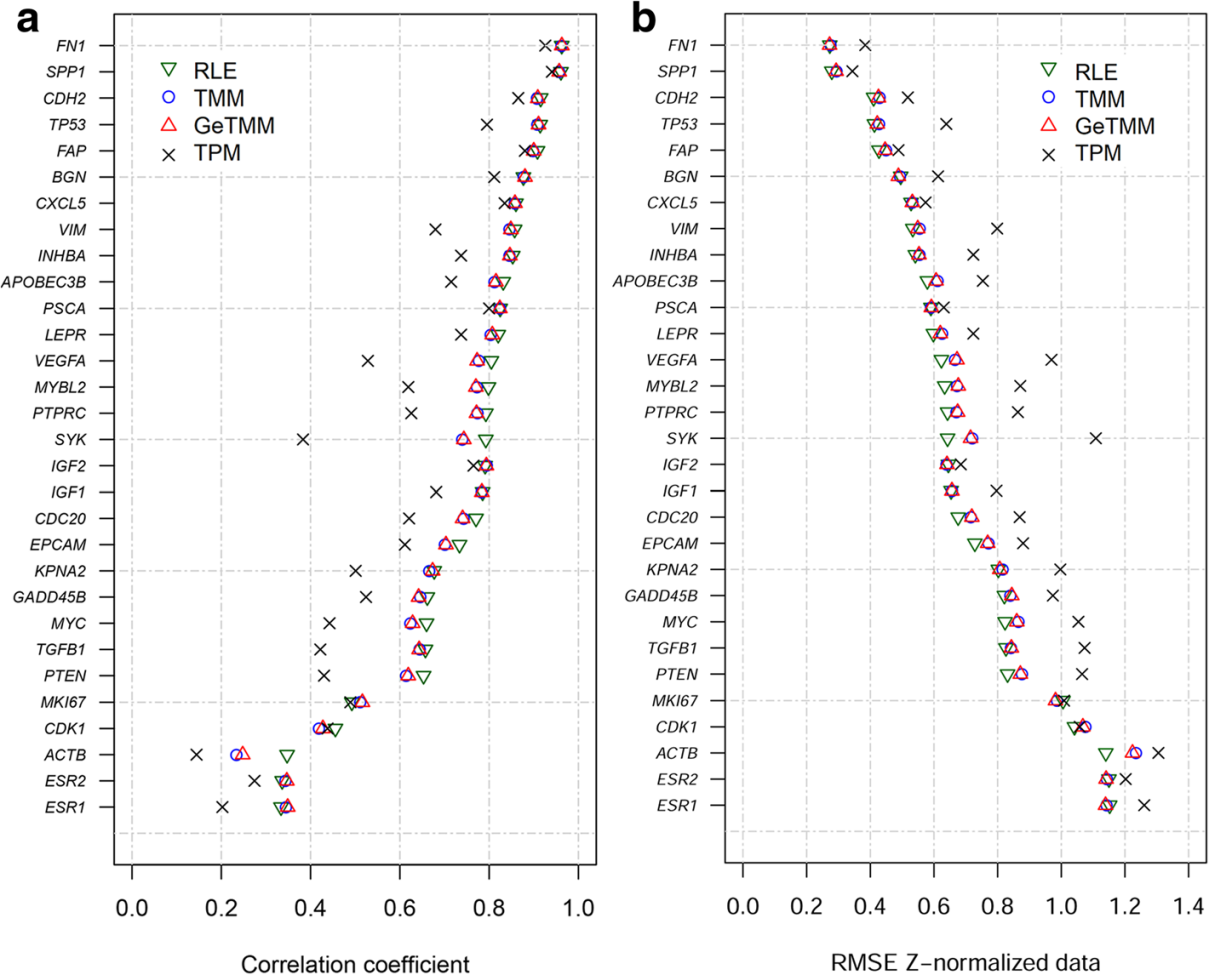
Correlation and RMSE to RT-qPCR data of 30 genes. **a** Correlation coefficients (x-axis) and **b** RMSE (x-axis) of 30 genes comparing RNA-seq normalization methods to RT-qPCR generated data

To evaluate how the different normalization methods affect downstream analysis, we measured the expression levels of 33 genes (of which 3 reference genes - *HMBS*, *HPRT1* and *TBP*) using RT-qPCR in the same RNA isolate as was used for sequencing. The RT-qPCR data were normalized using the reference genes and were considered as the gold standard to compare against. To assess the effect of the different normalization methods on intersample analysis, we correlated the normalized RNA-seq data of the 30 genes to the RT-qPCR levels over all samples (Fig. 3a, Additional file 5 and Additional file 6 for a detailed example). Overall, correlation coefficients for GeTMM were very comparable to the correlation coefficients for RLE and TMM normalized data, and higher than the correlation coefficients for TPM (Fig. 3a). For most genes, RLE had the highest correlation coefficients in absolute numbers, although the average and median difference with GeTMM showed very little difference in individual coefficients (0.014 and 0.008, respectively). Furthermore, no significant difference was observed between RLE, TMM and GeTMM normalized data (Mann-Whitney test, see Additional file 7) while TPM resulted in significantly lower coefficients compared to the other methods (*p* = 0.02, *p* = 0.04 and *p* = 0.03 for RLE, TMM and GeTMM, respectively). A Spearman’s rank correlation analysis on these data – to ascertain the influence of possible non-normally distributed expression data – showed the same results (Additional file 8). In addition, the RMSE of the methods compared to RT-qPCR data was calculated; to be able to do this we first standardized the data using Z-normalization, so that the data for each gene had a mean and SD of approximately 0 and 1, respectively. Without Z-normalization, meaningful interpretation of the RMSE would be obscured by the difference in expression ranges that the RNA-seq normalization methods have. RMSE values (Fig. 3b) of GeTMM, TMM and RLE were again very comparable, while TPM showed a general higher error.

**Fig. 4:**
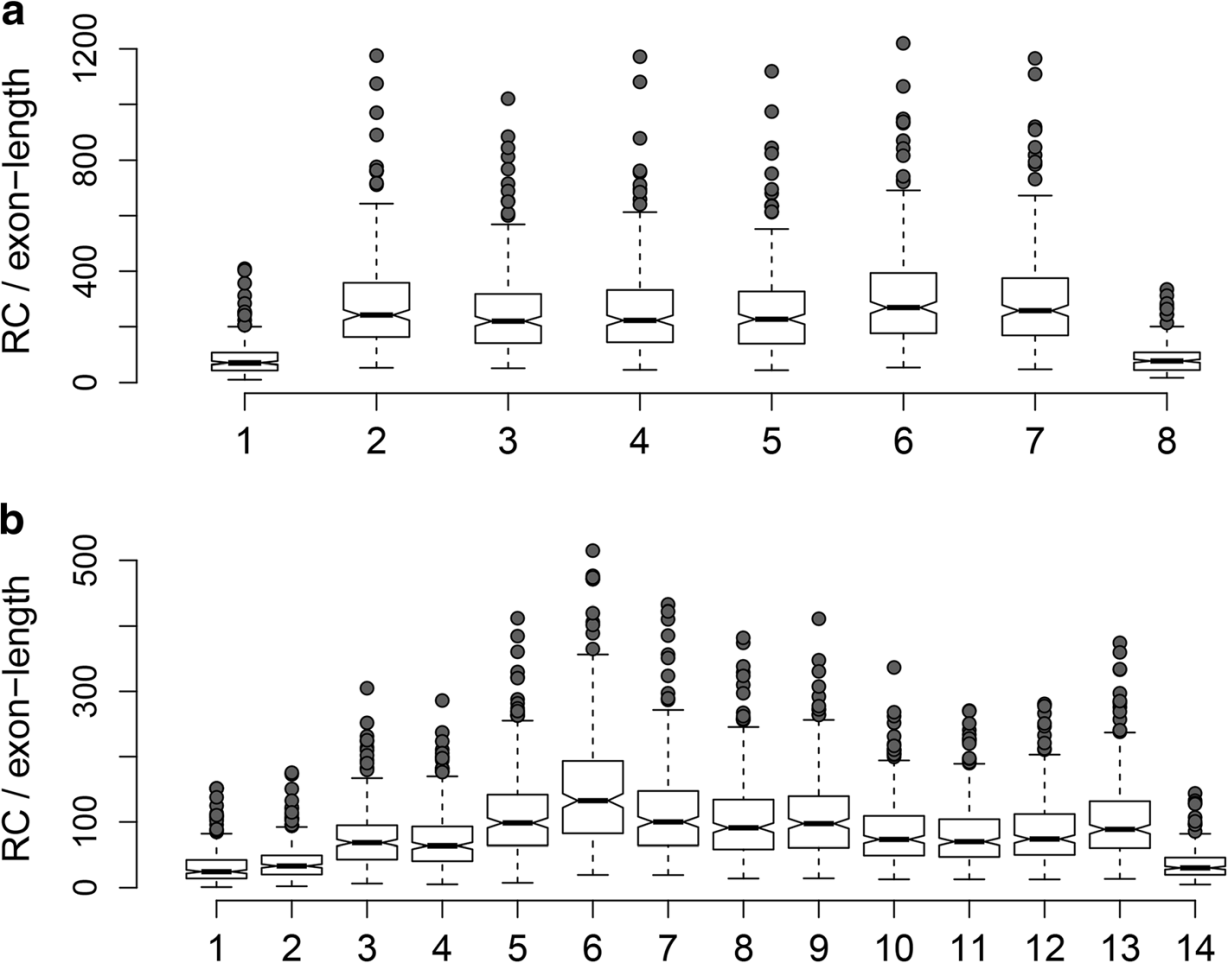
Boxplots of read counts per exon. **a** shows the expression levels in read counts per 100 bp for each exon in *CDK1 (*NB no additional normalization was performed). The whiskers extend to 1.5 IQR (interquartile range) above the third, or below the first quartile, with the median indicated by a horizontal line in the box. The notch indicates the 95% confidence interval of the median. **b** shows the same data for the *MKI67* gene

The aim of this part of the study was not to appraise the correlation coefficients obtained using the RT-qPCR data but to use the RT-qPCR data as benchmark so the RNA-seq normalization procedures could be compared with each other. Nonetheless, we further investigated the five genes that showed an *R* < 0.6; *MKI67*, *CDK1*, *ACTB, ESR1* and *ESR2*. The poor correlation of the latter 2 genes may be caused by the very low expression of these genes according to the RNA-seq data (median read count was just 22 for both *ESR1* and *ESR2*), indicating an insufficient sequencing depth for these genes. *ACTB* was the highest expressed gene of the 30 genes and had the lowest variance in 4 of 5 methods (0.25, 0.13, 0.16 and 0.16 for RT-qPCR, RLE, TMM and GeTMM, respectively), which may be the reason for the low correlation. For *CDK1* and *MKI67*, we re-analyzed all 263 samples to obtain the reads per exon. We observed a lower expression of exon 1 of *CDK1*, which may explain the poor correlation between the RT-qPCR and RNA-seq data as the RT-qPCR product spans exon 1 and 2 (Fig. 4a). A similar analysis for *MKI67* did not show the same effect; here the RT-qPCR assay spans exon 10 to 11, which both showed similar expression levels as the overall gene expression level (Fig. 4b). So unless transcript XM_006717864, which was the only truncated transcript of *MKI67* not covered by this RT-qPCR assay, is dominantly present in our sample cohort, we found no obvious explanation for this poor correlation.

### Comparison to RT-qPCR generated data: Intrasample analysis

**Fig. 5:**
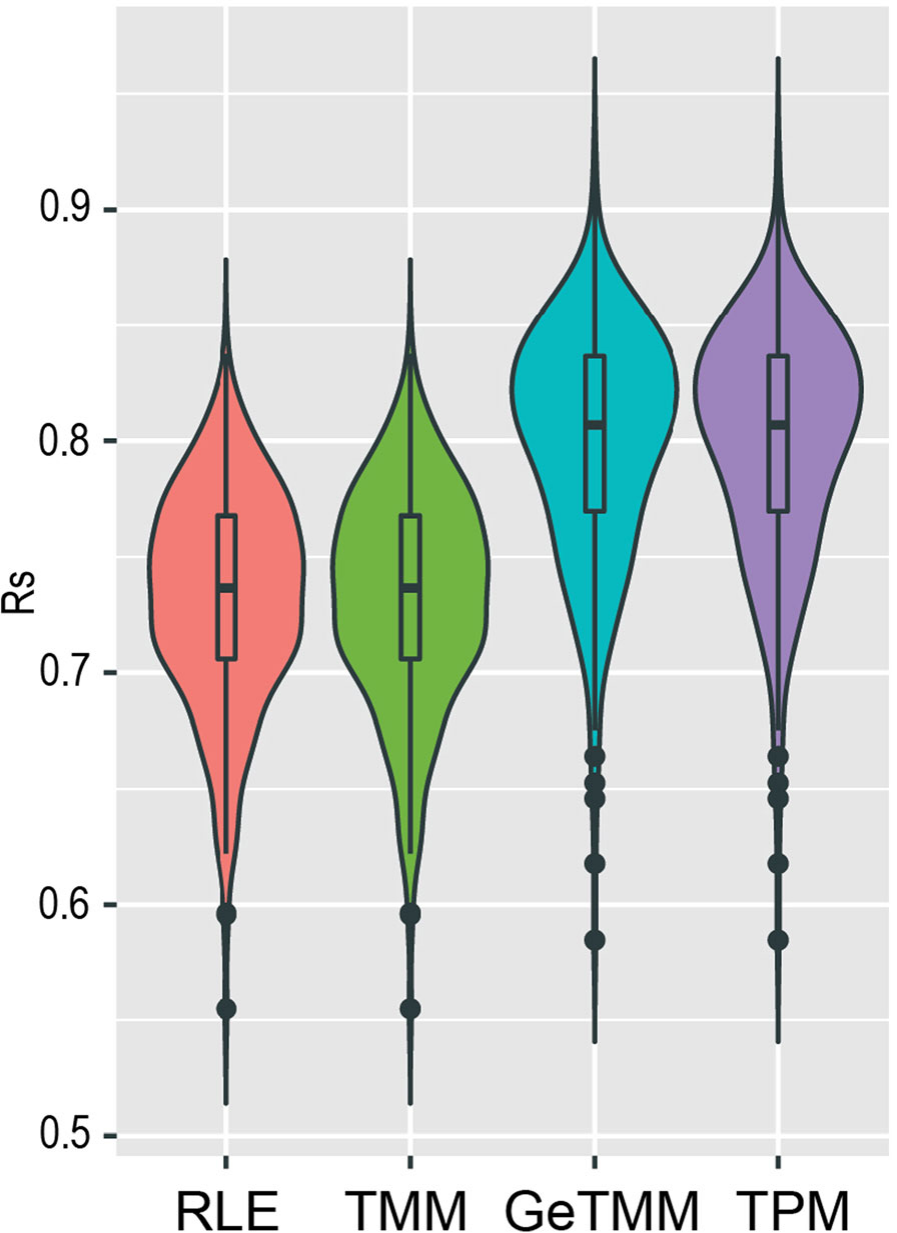
Violin plots of rank correlation by method. Spearman rank correlation coefficients of 263 samples by correlating each method with RT-qPCR generated data

Previously [20], RNA-seq normalization methods were compared to RT-qPCR data in the MicroArray Quality Control (MAQC) and Sequence Quality Control SEQC effort [21], using an alternative setup; 996 genes were measured in a single sample by RT-qPCR and these were correlated (Spearman’s rank) to gene-expression levels as measured by RNA-seq of the same sample. To mimic the SEQC results, we repeated the analysis with the RT-qPCR data of the 30 genes, and calculated a Spearman’s rank correlation coefficient between RT-qPCR and the different RNA-seq normalization methods for each of the samples, yielding 263 correlation coefficients per method (Fig. 5). GeTMM and TPM (the methods that include a gene length correction) both showed overall significant higher correlation to RT-qPCR data than RLE- and TMM-normalized data (Mann-Whitney *p* < 0.0001). GeTMM showed a higher correlation coefficient in 262 of the 263 cases.

### The performance of GeTMM is not affected by poor RNA quality

**Fig. 6:**
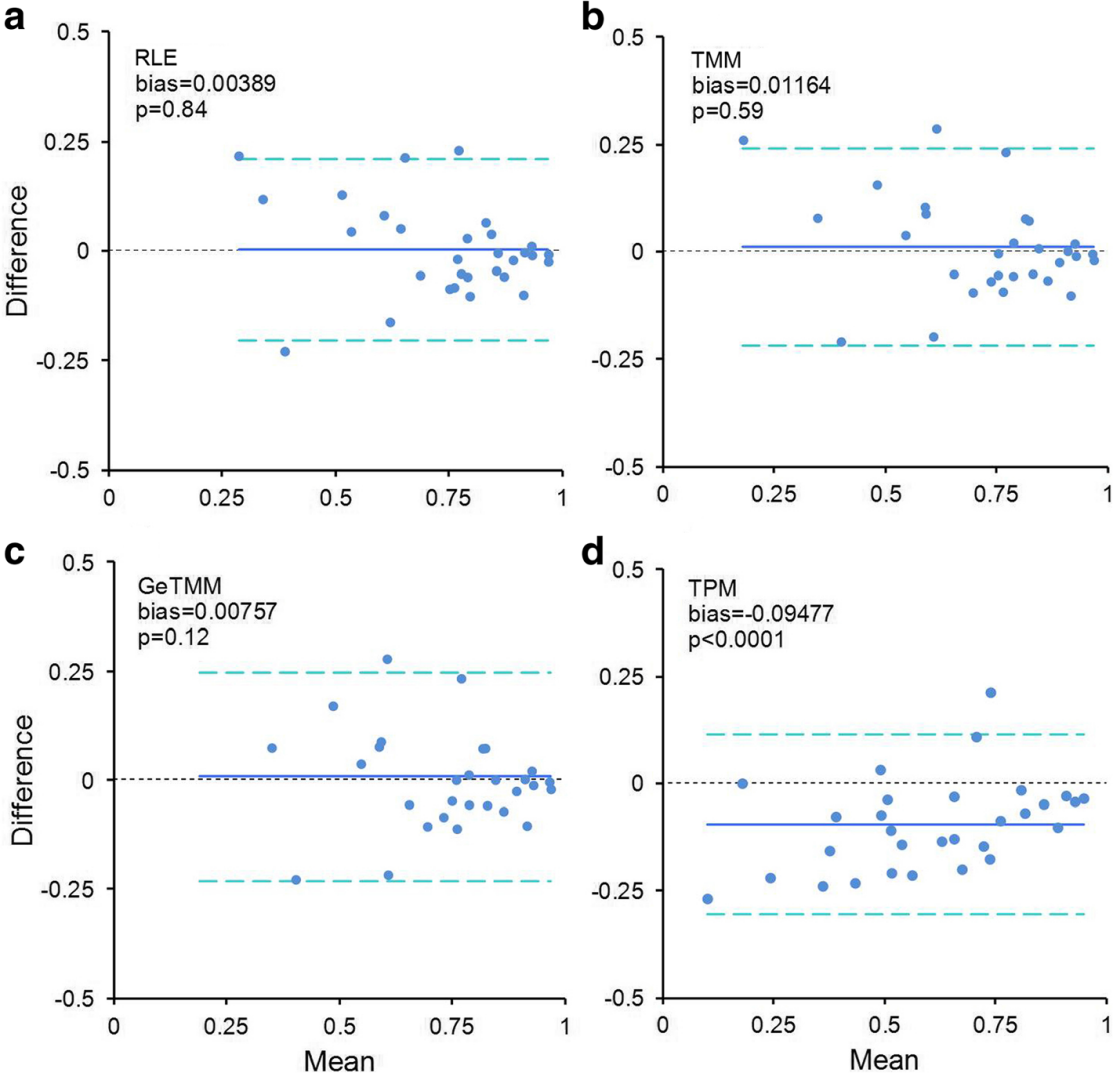
Bland-Altman plots comparing samples with high and low RIN values. **a-d**: for each normalization method, a group of 76 samples with low RIN values (< 7) was used to correlate expression data of 30 genes to RT-qPCR generated data. The same was performed for an equally sized high RIN sample group (> 9) and the correlation coefficients were compared. X-axis shows the mean correlation, the y-axis the difference (high RIN – low RIN). The blue line indicates the bias (mean of all differences), the dashed light-blue lines show the 95% limits of agreement, the dashed black line at zero is the identity line (indicating no difference). The *p*-value is derived from a one-sample t-test

Next, we repeated the intersample correlation analysis with RT-qPCR data for the 76 samples that had an RNA integrity (RIN) value < 7 after the cleanup procedure (median RIN 5.3), and compared these to an equally sized group of 76 samples with the highest RIN values (RIN > 9, median RIN 9.5). The median library size of the low RIN group was slightly lower at 5.58 million versus 6.52 million for the high RIN group (Mann-Whitney *p* = 0.02, see Additional file 9A). However, a principal component analysis using all expressed genes showed no separation of the low/high RIN groups, regardless of normalization method (Additional file 9B-E). Next, we correlated the RT-qPCR data to the RNA-seq data for each normalization method for the low and high RIN group separately, and compared the correlation coefficients between the groups. Figure 6a-d shows a Bland-Altman difference plot for the four methods with the mean bias and *p*-value (Student’s t-test under H0 that the difference is 0). Similar to the intersample comparison between RNA-seq and RT-qPCR in all samples, the result for GeTMM was similar to TMM and RLE normalized data, meaning the correlation coefficients were similar for the low and high RIN group. Normalization using TPM did result in significantly lower correlation coefficients in the high RIN group compared to the low RIN group (bias = − 0.09477, p < 0.0001), again indicating an advantage for GeTMM compared to TPM.

### GeTMM best resembles results of differential expression analysis using RT-qPCR

**Fig. 7:**
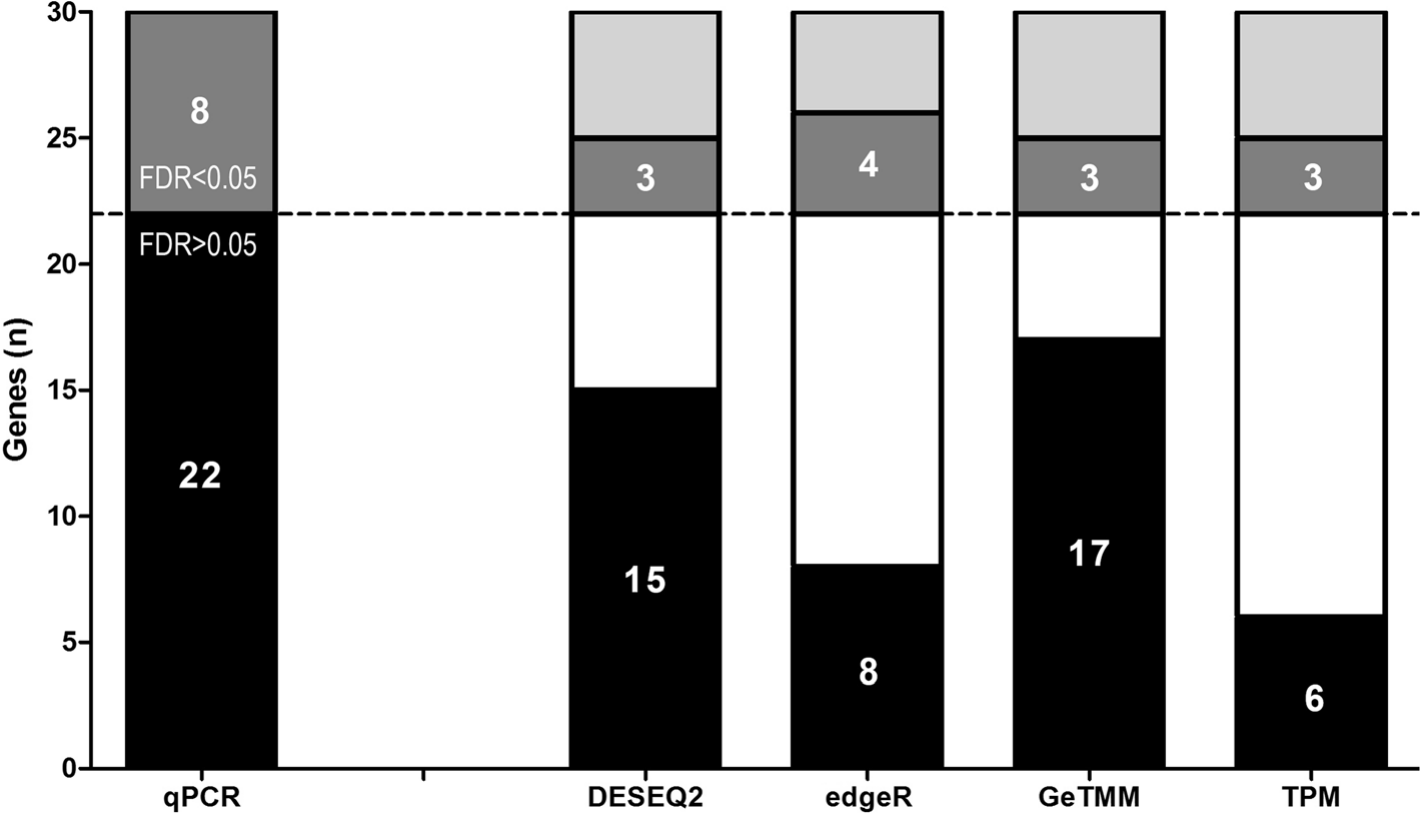
Number of DE genes between left and right sided tumors per normalization method. RT-qPCR generated data were used as benchmark, showing 8 genes with FDR < 0.05 (dark-grey) and 22 genes FDR > 0.05 (black). For the RNA-seq normalization methods, black indicate true negatives (FDR > 0.05, matches with RT-qPCR), white indicate false positives (FDR < 0.05, not matching RT-qPCR), grey indicate true positives (FDR < 0.05, matches RT-qPCR) and light-grey indicate false negatives (FDR > 0.05, not matching RT-qPCR)

The correlation of the different normalization methods to RT-qPCR data already showed that GeTMM performed equivalent to TMM and RLE, but outperformed TPM. To further study the effect of the different normalization methods on an intersample analysis in a biological relevant context, the genes in left sided and right sided colon tumors were examined for differential expression, since tumors in the left and right hemicolon are known to be biologically different. In short, right-sided tumors are frequently hypermethylated, hypermutated, microsatellite instable and *BRAF*-mutated while left-sided tumors are frequently microsatellite stable and frequently carry an APC and *KRAS*-mutation [22]. This characteristic roughly divided our cohort in half (48% left-sided and 52% right-sided). We evaluated all 30 genes in the RT-qPCR data set by a standard t-test and after multiple testing correction (Benjamini-Hochberg) 8 genes showed an FDR < 0.05: *MYBL2, MYC, EPCAM, SYK, APOBEC3B, SPP1, CDK1* and *IGF1*. Next, to check if the RNA-seq normalization methods showed differences in the amount of removal/compression of relevant biological variation, we calculated the Signal-to-Noise ratio (SNR) for these 8 genes. Again, GeTMM performed similar to TMM and RLE normalized data, showing very comparable SNRs, but outperformed TPM (see Additional file 10). Up to now, we used DESeq2 and edgeR normalized data (RLE and TMM, respectively), however, these methods are intended for both normalization and identification of DE genes. Each uses a statistical test that was designed for use in the respective package (a Wald test and exact test for DESeq2 and edgeR, respectively). Thus, in order to evaluate the performance of GeTMM in identifying DE genes in comparison with DESeq2 and edgeR, the statistical tests implemented by edgeR and DESeq2 were run on the respective data sets, while for TPM and GeTMM data, Student’s t-tests were used on the 30 genes. Figure 7 shows the results of comparing FDR adjusted *p*-values by normalization method. Out of the 22 genes that were not DE according to the RT-qPCR data, GeTMM had the lowest number of ‘false positives’ (5/22) compared to edgeR (14/22), DESeq2 (7/22) and TPM (16/22). The recall was similar for all methods (4 out of 8 for edgeR, and 3 out of 8 for the other methods). When analyzing TMM (edgeR) and RLE (DESeq2) normalized data with a t-test, recall of edgeR dropped to 3 genes while DESeq2 recalled 4 genes. Both edgeR and DESeq2 called 5 genes as ‘false-positives’ (the same 5 genes GeTMM calls significant).

### Gene length correction benefits TMM in the Oncotype DX® recurrence score

**Fig. 8:**
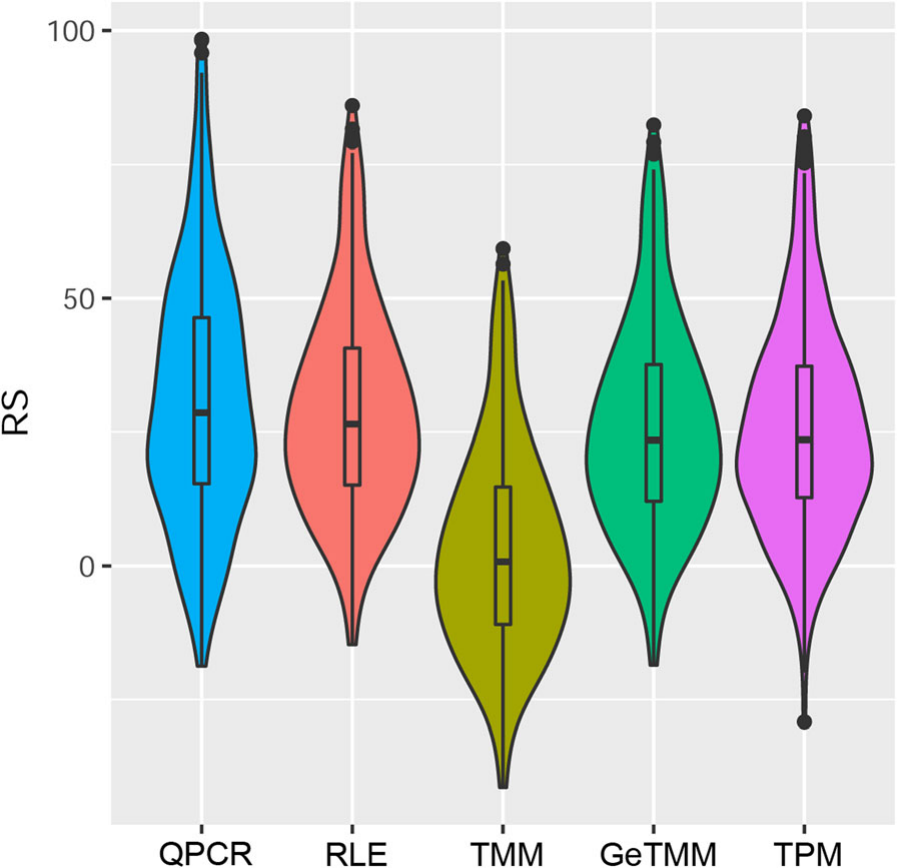
Violin plots of the recurrence score. The Onco*type* DX ® Recurrence Score (RS) of 263 samples by method

An often-used tool to estimate risk of recurrence in colon cancer is the Recurrence Score (RS) algorithm of Onco*type* DX® [14], which uses a 7 cancer-gene panel. The RS was calculated for all samples, based on the RT-qPCR data as well as the RNA-seq normalized datasets (Fig. 8). The distribution of the RT-qPCR generated scores are very similar to the scores generated using RNA-seq, except for the TMM derived RS. The overall lower scores will impact the RS evaluation, as the original RS is scaled such that negative scores will be set to zero. Using TMM, 41% of patients (*n* = 109) would receive this score. Clearly GeTMM, which uses gene length correction on top of edgeR normalization, improves the range and distribution of the RS scores.

### Gene length correction impacts CMS prediction

Finally, the CMS classification was determined for each sample using data normalized by the different methods [13]. In this classification five possible groups are predicted: CMS1–4 and mixed/indeterminate. The type of classification is based on correlation of gene-signatures specific for each subtype to an individual sample, making this an intrasample-type analysis. Perfect agreement in the predicted CMS groups was seen between RLE and TMM normalized data (both without gene length correction), and between TPM and GeTMM (both with gene length correction). However, gene length correction had a considerable impact on the prediction of the CMS groups: 40 samples (15.2%) were predicted in a different group when comparing TMM and GeTMM (Table 1).

**Table 1:** Predicted CMS group by normalization method

	GeTMM					
**TMM**	CMS1	CMS2	CMS3	CMS4	Mixed/indeterminate	Total
CMS1	**46**	0	0	0	7	53
CMS2	0	**127**	0	0	5	132
CMS3	0	0	**23**	0	0	23
CMS4	0	1	0	**5**	4	10
Mixed/indeterminate	3	14	6	0	**22**	45
Total	49	142	29	5	38	263

## Discussion

The current study showed that GeTMM performed equivalent in intersample analyses to two commonly used and best performing in several RNA-seq normalization aspects – RLE (used by DESeq2) and TMM (used by edgeR, both do not use gene length correction) [6–8], while outperforming these methods in intrasample comparisons. Therefore, GeTMM generates a normalized data set directly suited for multiple endpoints. The effects of the different methods on the distribution of the gene expression data, samples with different RNA quality, subtype-classification, recurrence score, recall of DE genes, RMSE analysis and correlation to RT-qPCR data were assessed in a large cohort of real (i.e. not simulated) data, obtained from 263 primary colon tumors. Importantly, the current study focused on the application of RNA-Seq data for differential expression analysis between and within samples, thus not covering other applications such as the detection of fusion events, variant analysis and gene isoforms [23]. With regard to the latter, the normalization methods used in this study including GeTMM were not developed to distinguish possible isoforms, which requires estimating expression on a transcript level using more complex models and different statistics [10, 24, 25]. Thus, the investigated normalization methods may not be fully appropriate for such transcript level analyses.

The effect of gene length correction on downstream analysis is more important than it seems at first, when realizing that several frequently used standard analyses are vulnerable to gene length induced bias. Besides the theoretical example stated in the introduction, another example is e.g. in breast cancer, wherein the AIMS [26] method was developed to obtain a truly independent single sample classifier to robustly call molecular subtypes. Herein, subtype-specific genes are evaluated within each sample; e.g. when *GRB7* (a 532 bp transcript) is higher expressed than *BCL2* (a 239 bp transcript), it adds to the evidence for a HER2 subtype [26]. Without correcting for gene length, this prediction method will not work as intended on RNA-seq data as *GRB7* read counts will be about 2-fold higher compared to the *BCL2* read counts, when both genes are expressed at equal levels. Evaluating these intrasample-type analyses in the current study, GeTMM and TPM produced significantly better results compared to data normalized by TMM (edgeR) and RLE (DESeq2) when correlating a set of genes measured by different methods within the same sample. A similar sort of analysis had been performed previously [20] using the data available from the MicroArray Quality Control (MAQC) effort, wherein more genes were measured by RT-qPCR, but only using two samples. In our study we used 263 samples, thus capturing the biological variation of gene expression levels much better. Regarding clinical applicability, this study showed that gene length correction influences the prediction of the subtypes (CMS) of colorectal cancer [13]. Given the methodology of the CMS classifier, where the gene expression data of a single sample are correlated to a centroid of a set of genes that are specific to each of the 4 CMS groups, it makes more sense to use a normalization that includes a gene length correction, to avoid under- or overestimating the true expression levels of genes within a sample. Of note, we do not claim to predict the true CMS classification, but assuming that the GeTMM classification reflects a more reliable prediction, 23 samples would change from a CMS group to mixed/indeterminate using a method without gene length correction, and 1 sample would change from CMS2 to CMS4. In calculating the recurrence score (Onco*type* DX®) edgeR showed an overall much lower distribution and assigned almost half of the patients below a zero score. This was remedied by including a gene length correction (thus yielding GeTMM), resulting in scores very comparable and in the same range as the RT-qPCR generated scores. This illustrates the importance of using a normalization method like GeTMM, that results in a data set that is suited for both intersample as well as intrasample analyses.

Several metrics were used to evaluate the normalization methods, summarized in Table 2. In general, TPM is not sufficient to correct for between-sample differences. This echoes previously reported results using RPKM and FPKM normalization [5, 6, 11, 12], and it is reasonable to conclude that normalization by library size alone must be abandoned as viable method to detect DE genes between samples. RLE and TMM normalized data differed only slightly with respect to distribution, correlation and RMSE to RT-qPCR and sensitivity to RNA quality, and not at all with regard to the CMS classification. However, the statistical test that edgeR employs seemed overly optimistic in identifying DE genes while DESeq2’s statistical test is more conservative, a difference that was also observed by others [8]. Given the strong similarities between the data after normalization with RLE and TMM, the differences in the reported DE genes are more likely a result of differences in the statistical tests employed by both methods than by the normalization itself. This was confirmed by using a t-test for all normalization methods, showing very comparable results; thus, GeTMM performed similar to edgeR and DESeq in the intersample analysis of identifying DE genes.

**Table 2:** Summary of results

Normalization Method	Gene length correction	Distribution per sample	Influence of RIN on correlation	Intersample correlation	Intrasample correlation
RLE (DESeq2)	no	bimodal	no bias	++	+
TMM (edgeR)	no	bimodal	no bias	++	+
TPM	yes	normal	bias	–	++
GeTMM	yes	normal	no bias	++	++

A ‘-’ indicates a relative poor performance for the given criterion, and increasing performance is indicated by ‘+’ and ‘++’

The analyses using subsets of samples with a low or high RIN value showed remarkably little difference in correlation to RT-qPCR generated data. It appears that samples with a low RIN value may yield sequencing data suitable for expression analyses. Still, this conclusion is drawn from a single correlation analysis and may be very specific to the entire protocol that was used (RNA isolation, library prep etc.) and may therefore not be applicable to all studies and protocols. Still, a-priori disregarding samples with a low RIN value for sequencing could prove wasteful, though it is prudent to perform a robust QC on the generated sequencing data to spot failed samples.

Lastly, this study uses RT-qPCR as standard so the RNA-seq normalization methods could be compared with each other. RT-qPCR is known for its precise and reproducible measurements and may have a bigger dynamic range compared to the usual coverage of sequence data. The downside is that RT-qPCR measures just a small part of the gene, may miss or be affected by splice-variants, and can be affected by SNPs in the primer regions. In that respect, the RNA-seq generated data may be nearer the mark of the actual expression level of a gene. In the future, RNA-seq may replace RT-qPCR as the gold standard for expression data, provided a well-founded normalization method is used.

## Conclusions

This study shows that GeTMM produces a versatile normalized RNA-seq data set, appropriate for both inter- and intrasample comparisons. This quality of GeTMM should further enhance the capacity of RNA-seq as a solid method to explore and compare gene expression profiles, and thus may become increasingly interesting in the current era of data sharing efforts.

## Additional files

Additional file 1: Impact of gene length correction on correlation. Simulated expression data of 10 genes in 2 samples. Correlation based on read counts show different results after correcting for gene length. RPK indicates reads per kilobase. (PDF 189 kb)

Additional file 2: Correlation RIN BioAnalyzer vs MultiNA. RIN values as measured by the Bioanalyzer (Agilent) were compared to the 28S/total concentration as measure by the MultiNA in a training set of 60 cases (A). The resulting trend line was validated in an independent cohort (B) of 73 cases. (PDF 199 kb)

Additional file 3: Details on the RT-qPCR assays. (XLSX 14 kb)

Additional file 4: Details on the settings for the STAR algorithm and R commands to obtain normalized data. (DOCX 13 kb)

Additional file 5: Correlation coefficients for each method compared to RT-qPCR. (XLSX 10 kb)

Additional file 6: Expression of *PSCA* and comparison of several RNA-Seq normalization methods. (TIFF 9492 kb)

Additional file 7: Comparison correlation coefficients by method. Boxplots show correlation coefficient of 30 genes, comparing 4 methods to RT-qPCR generated data. *P*-values are derived from the Mann-Whitney test. (PDF 3492 kb)

Additional file 8: Spearman’s correlation to RT-qPCR data of 30 genes. Correlation coefficients (x-axis) of 30 genes comparing RNA-seq normalization methods to RT-qPCR generated data. (PDF 618 kb)

Additional file 9: Library size and PCA plots by RIN. A shows the library size (log10) in samples with low RIN values (RIN < 7) or high RIN (> = 9). B-E show PCA plots, colored by samples with low RIN (red) or high RIN (blue), by normalization method. (PDF 2842 kb)

Additional file 10: Signal-to-Noise Ratios. (XLSX 9 kb)

## Abbreviations

CMS: Consensus Molecular Subtypes
DE: Differentially Expressed
FDR: False Discovery Rate
FPKM: Fragments Per Kilobase per Million reads
GeTMM: Gene length corrected TMM
MAQC: MicroArray Quality Control
RIN: RNA Integrity Number
RLE: Relative Log Expression
RPK: Reads Per Kilobase
RPKM: Reads Per Kilobase per Million reads
RS: Recurrence Score
RT-qPCR: Reverse Transcriptase-quantitative Polymerase Chain Reaction
SEQC: Sequence Quality Control
TMM: Trimmed Mean of M-values
TPM: Transcript Per Million
