# Supplementary material for: Gene length corrected trimmed mean of M-values (GeTMM) processing of RNA-seq data performs similarly in intersample analyses while improving intrasample comparisons
Source: BMC Bioinformatics. 2018 Jun 22;19:236. doi: 10.1186/s12859-018-2246-7 (PMC6013957; doi:10.1186/s12859-018-2246-7)

		Readcounts		RPK	
gene	length (kb)	sample1	sample2	sample1	sample2
gene1	0.25	70	78	280.00	312.00
gene2	0.5	251	157	502.00	314.00
gene3	0.75	201	235	268.00	313.33
gene4	1	183	314	183.00	314.00
gene5	1.25	339	392	271.20	313.60
gene6	1.5	572	471	381.33	314.00
gene7	1.75	480	549	274.29	313.71
gene8	2	754	628	377.00	314.00
gene9	2.25	759	706	337.33	313.78
gene10	2.5	904	785	361.60	314.00

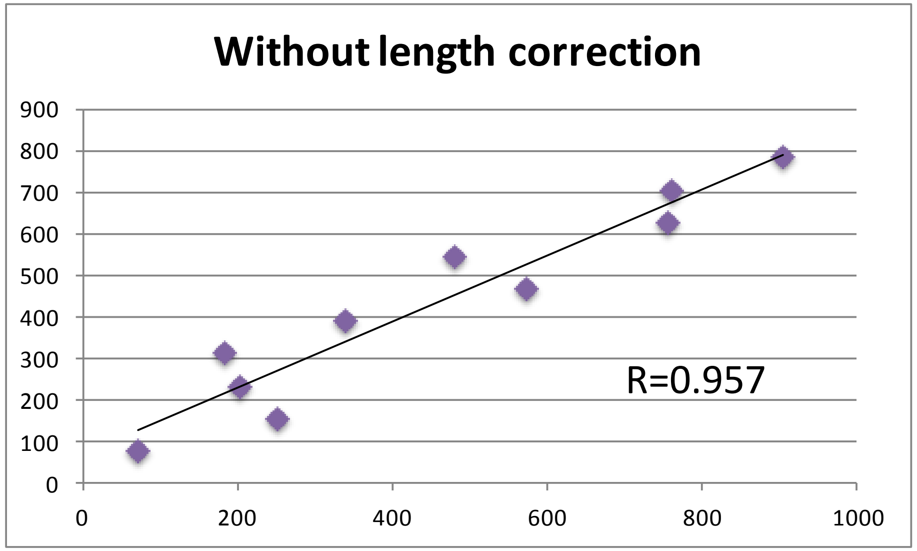

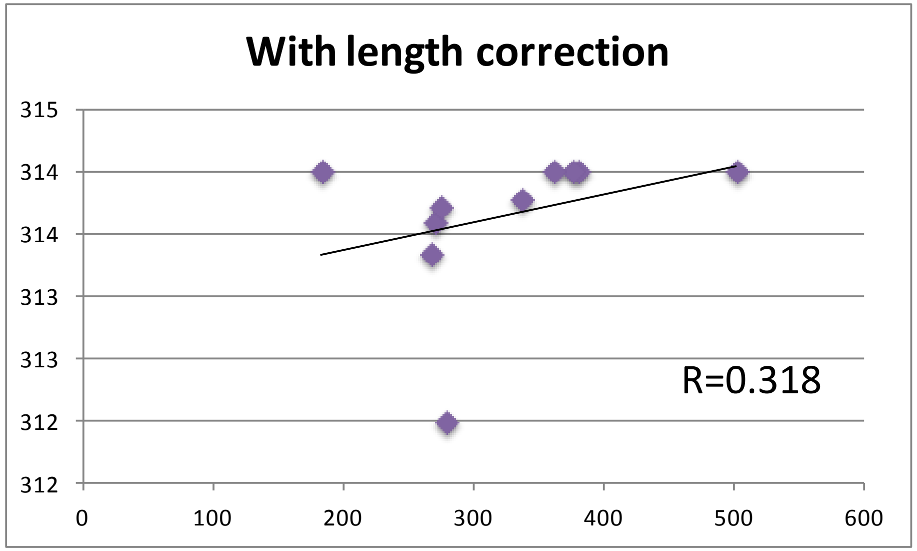

Supplement: Supplementary file 1 — Impact of gene length correction on correlation. Simulated expression data of 10 genes in 2 samples. Correlation based on read counts show different results after correcting for gene length. RPK indicates reads per kilobase. (PDF 189 kb) [file 12859_2018_2246_MOESM1_ESM.pdf]
