# Supplementary material for: Gene length corrected trimmed mean of M-values (GeTMM) processing of RNA-seq data performs similarly in intersample analyses while improving intrasample comparisons
Source: BMC Bioinformatics. 2018 Jun 22;19:236. doi: 10.1186/s12859-018-2246-7 (PMC6013957; doi:10.1186/s12859-018-2246-7)

TRAINING: [y=RIN BioAnalyzer, x=RIN MultiNA Conc. 28S/Total Conc]

Training set: Total RNA isolated with RNA-B from 60 freshly frozen breast tumor tissues

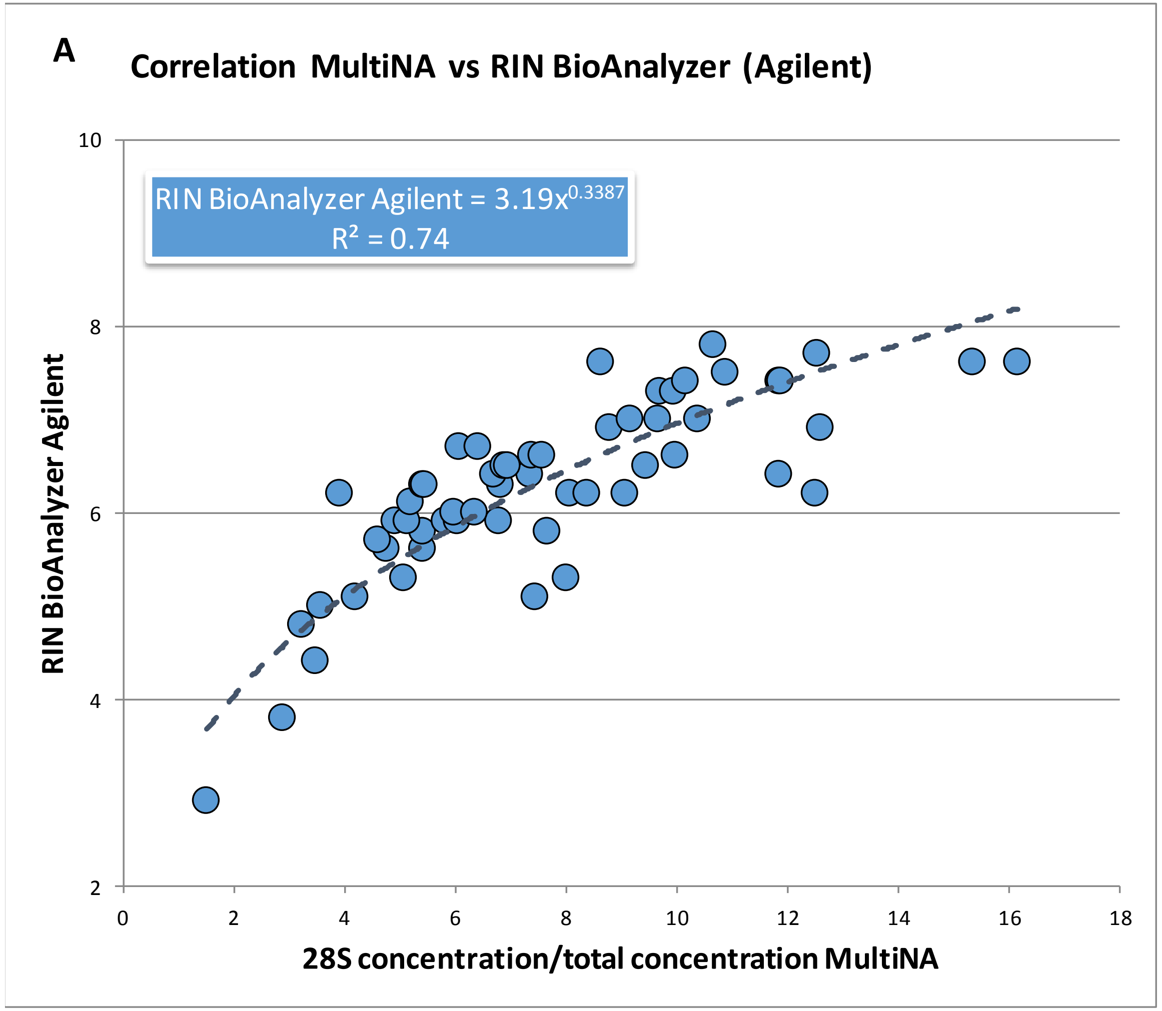

VALIDATION: [RIN MultiNA =3.19 * (28S Conc./total RNA Conc. * 100)$^{0.3387}$

Testing set: Total RNA isolated with RNA-B from 73 independent freshly frozen breast tumor tissues

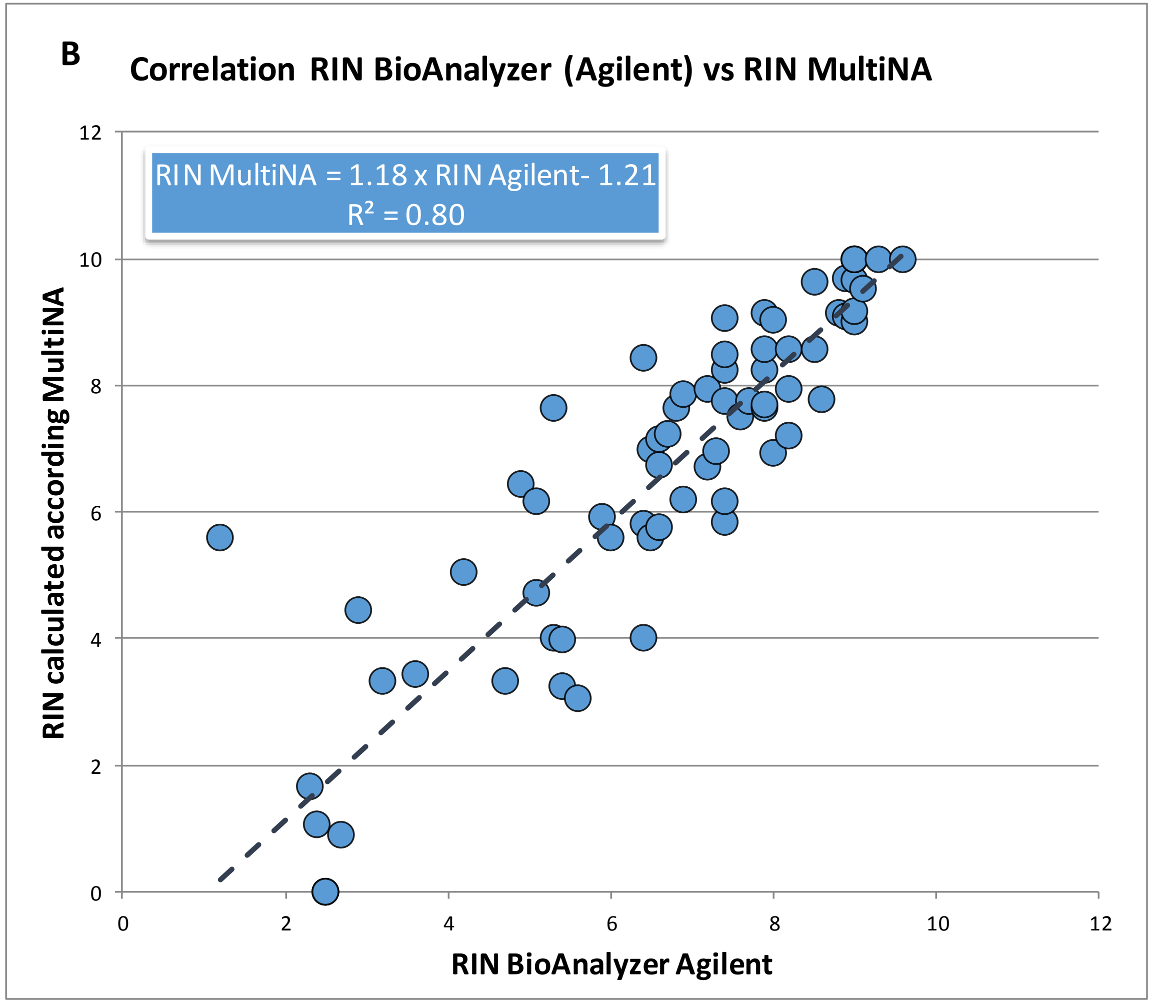

Supplement: Supplementary file 2 — Correlation RIN BioAnalyzer vs MultiNA. RIN values as measured by the Bioanalyzer (Agilent) were compared to the 28S/total concentration as measure by the MultiNA in a training set of 60 cases (A). The resulting trend line was validated in an independent cohort (B) of 73 cases. (PDF 199 kb) [file 12859_2018_2246_MOESM2_ESM.pdf]
