# Supplementary material for: Gene length corrected trimmed mean of M-values (GeTMM) processing of RNA-seq data performs similarly in intersample analyses while improving intrasample comparisons
Source: BMC Bioinformatics. 2018 Jun 22;19:236. doi: 10.1186/s12859-018-2246-7 (PMC6013957; doi:10.1186/s12859-018-2246-7)
Supplement: Supplementary file 4 — Details on the settings for the STAR algorithm and R commands to obtain normalized data. (DOCX 13 kb) [file 12859_2018_2246_MOESM4_ESM.docx]

**Additional file 4**

STAR algorithm

The STAR algorithm (version 2.4.2a) was used to align the RNA-seq data on the GRCh38 reference genome. Settings were:

--outSAMstrandField intronMotif

--outFilterIntronMotifs RemoveNoncanonicalUnannotated

--chimSegmentMin 12

--chimJunctionOverhangMin 12

--alignSJDBoverhangMin 10

--alignMatesGapMax 200000

--alignIntronMax 200000

--outSAMtype BAM SortedByCoordinate

--outSAMunmapped Within

--alignEndsType Local

--chimOutType WithinBAM

--twopassMode Basic

--twopass1readsN -1

--quantMode GeneCounts

Normalization

The raw readcount matrix (tab-delimited text file) was used, in which the first column holds the geneID from Ensembl that are used as row names in data matrix (x) in R, the second column of the text file (thus the first column in x) holds the gene length in kb and the remaining columns contain read counts of each sample.

# calculate RPK

rpk <- (x[,2:ncol(x)]/x[,1])

# remove length col in x

x <- x[,-1]

# for normalization purposes, no grouping of samples

group <- c(rep("A",ncol(x)))

#EdgeR

x.norm.edger <- DGEList(counts=x,group=group)

x.norm.edger <- calcNormFactors(x.norm.edger)

norm.counts.edger <- cpm(x.norm.edger)

#GeTMM

rpk.norm <- DGEList(counts=rpk,group=group)

rpk.norm <- calcNormFactors(rpk.norm)

norm.counts.rpk_edger <- cpm(rpk.norm)

#TPM

tpm = rpk

for (i in 1:ncol(rpk) ) {

tpm[,i] <- rpk[,i]/(sum(rpk[,i])/1e6)

}

#DESeq2

# no group & no design implemented

colData = data.frame(group)

rownames(colData)=colnames(x)

dds<-DESeqDataSetFromMatrix(countData=x,colData=colData, design=~ 1)

dds <- estimateSizeFactors(dds)

sizefact <- sizeFactors(dds)

norm.counts.deseq <- counts(dds, normalized=TRUE)

After processing, read counts were log2-transformed (setting genes to NA when having 0 read counts).
