# Supplementary figures and images for: Gene length corrected trimmed mean of M-values (GeTMM) processing of RNA-seq data performs similarly in intersample analyses while improving intrasample comparisons
Source: BMC Bioinformatics. 2018 Jun 22;19:236. doi: 10.1186/s12859-018-2246-7 (PMC6013957; doi:10.1186/s12859-018-2246-7)

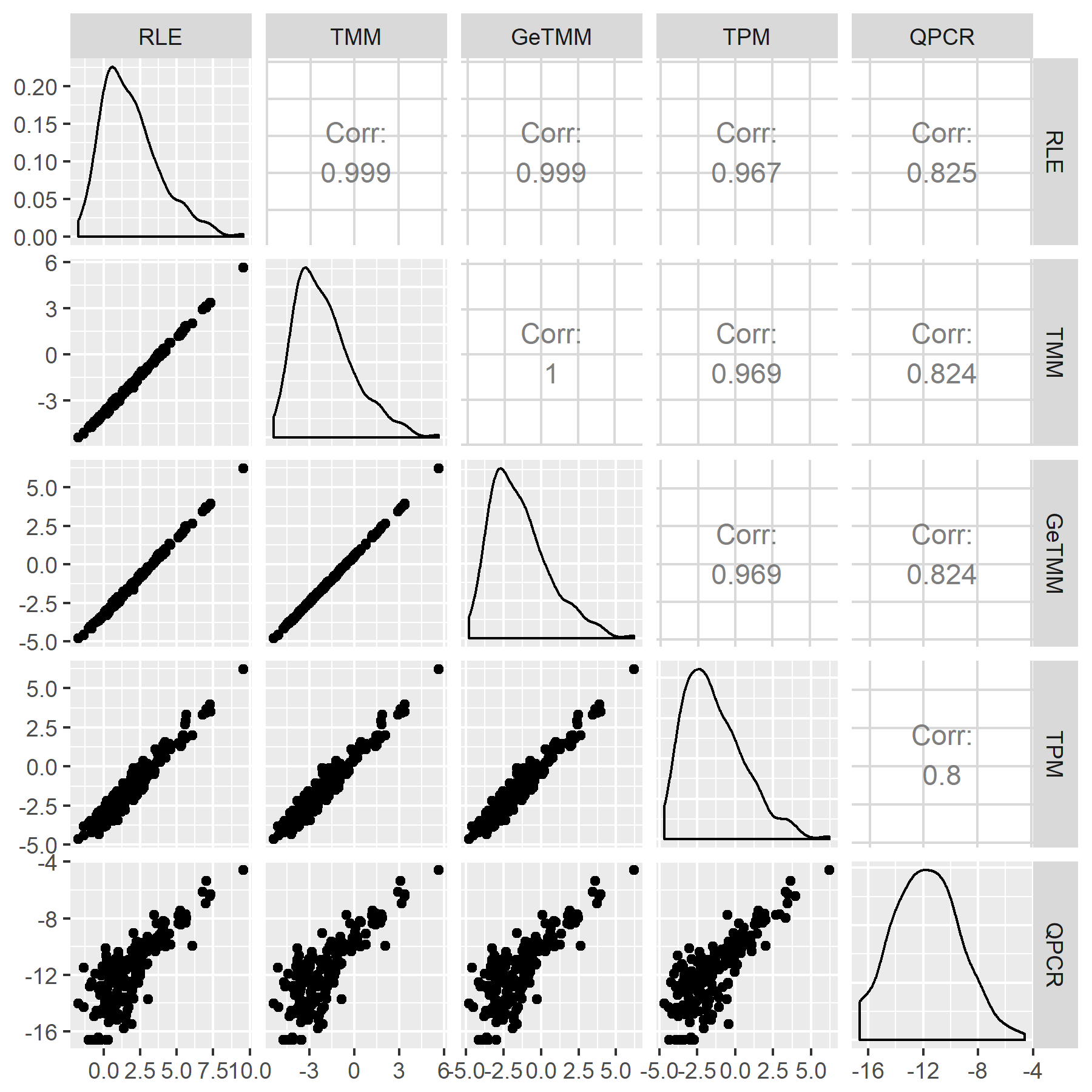

Supplement: Supplementary file 6 — Expression of PSCA and comparison of several RNA-Seq normalization methods. (TIFF 9492 kb) [file 12859_2018_2246_MOESM6_ESM.tiff]

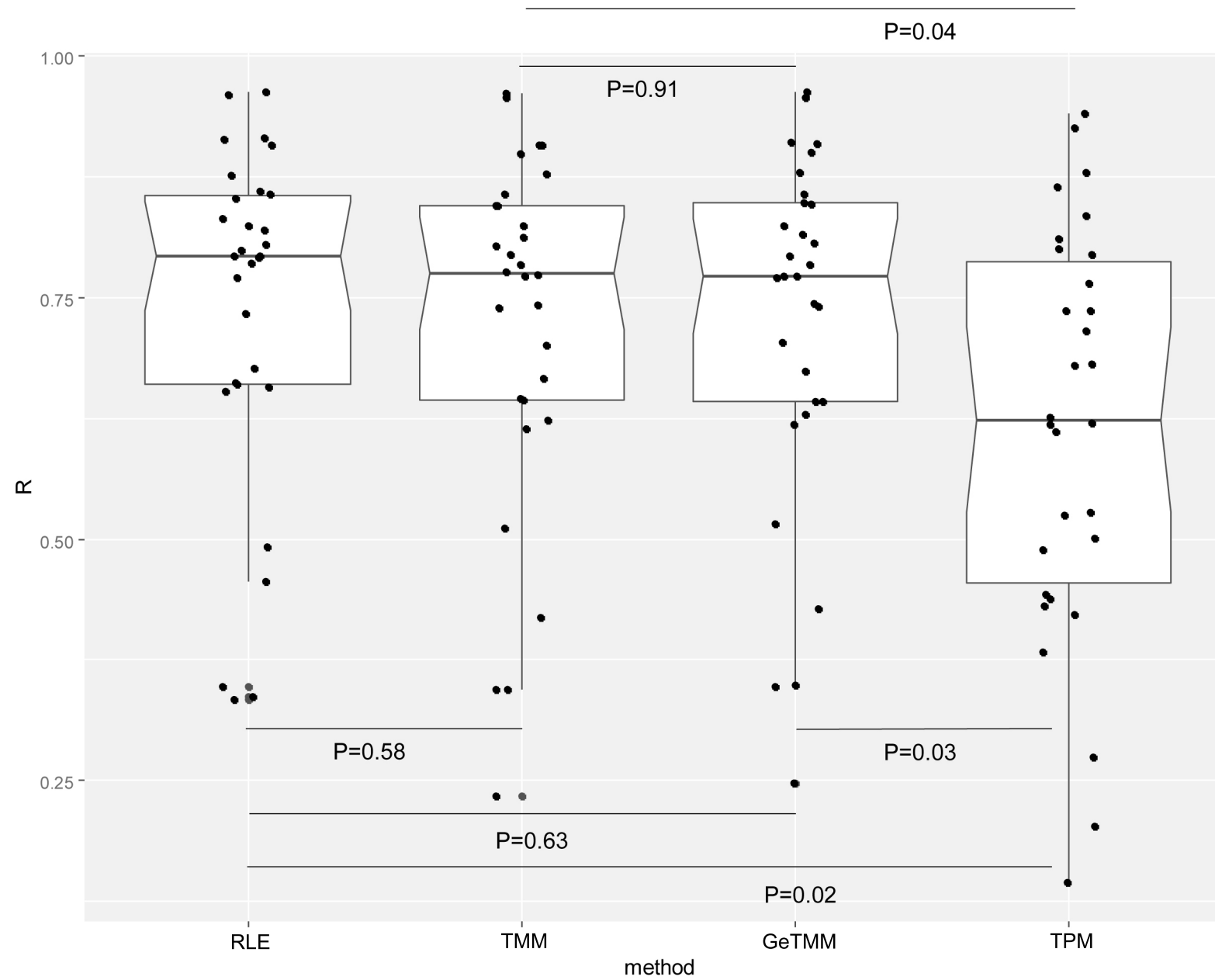

Supplement: Supplementary file 7 — Comparison correlation coefficients by method. Boxplots show correlation coefficient of 30 genes, comparing 4 methods to RT-qPCR generated data. P-values are derived from the Mann-Whitney test. (PDF 3492 kb) [file 12859_2018_2246_MOESM7_ESM.pdf]

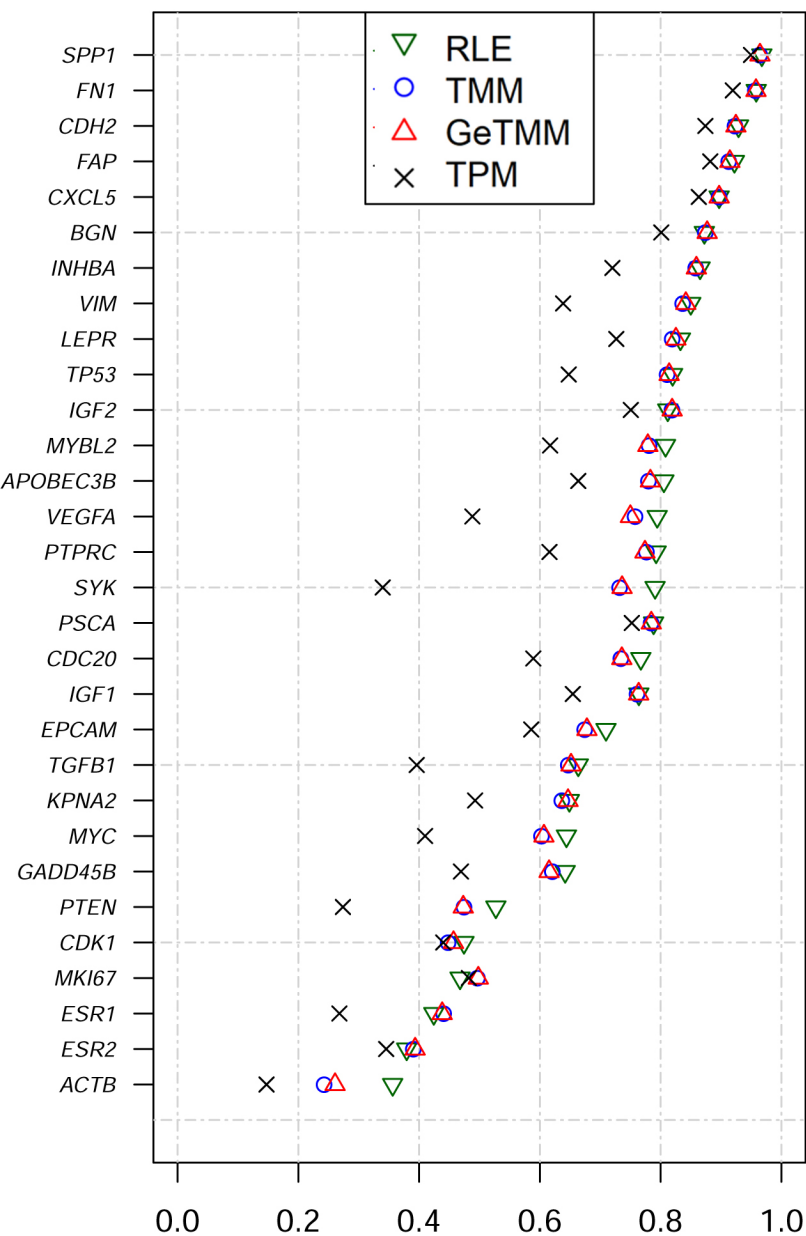

Spearman Correlation Coefficient

Supplement: Supplementary file 8 — Spearman’s correlation to RT-qPCR data of 30 genes. Correlation coefficients (x-axis) of 30 genes comparing RNA-seq normalization methods to RT-qPCR generated data. (PDF 618 kb) [file 12859_2018_2246_MOESM8_ESM.pdf]

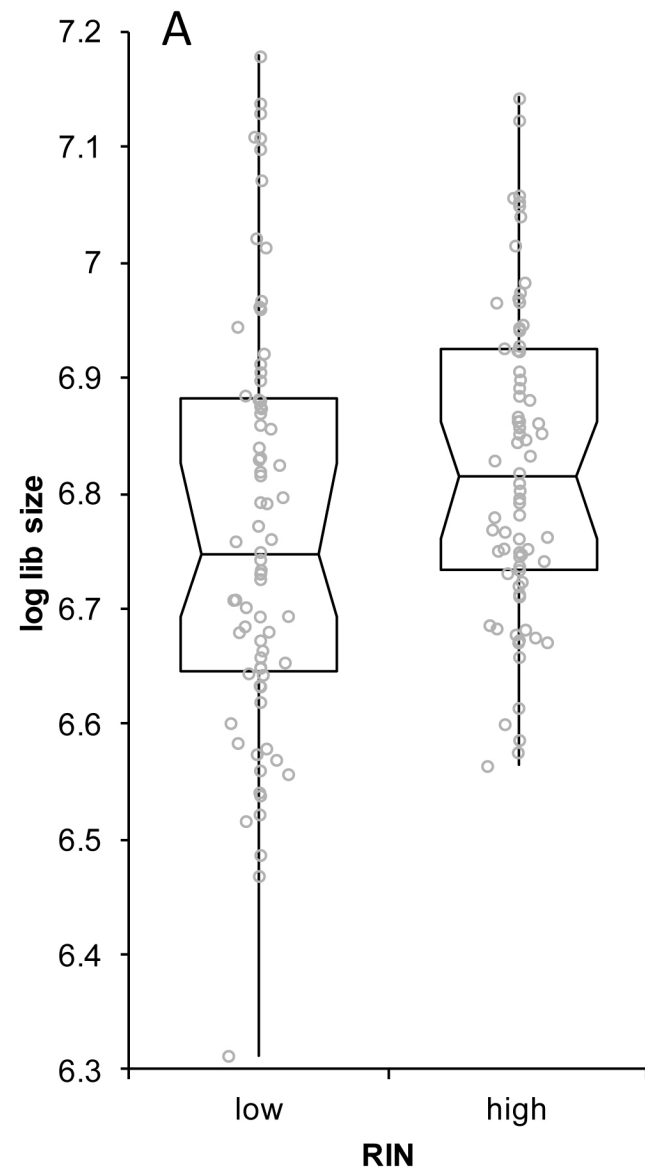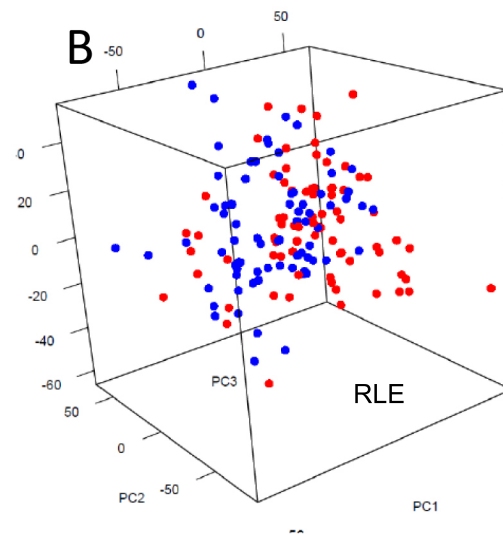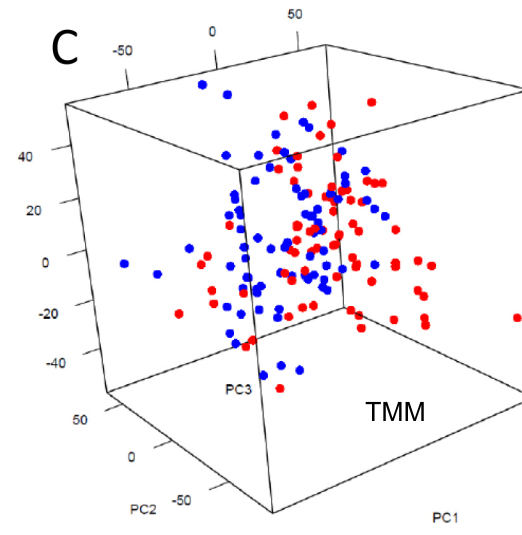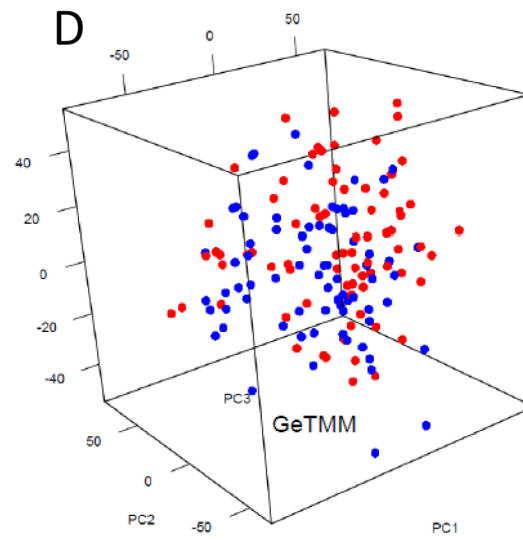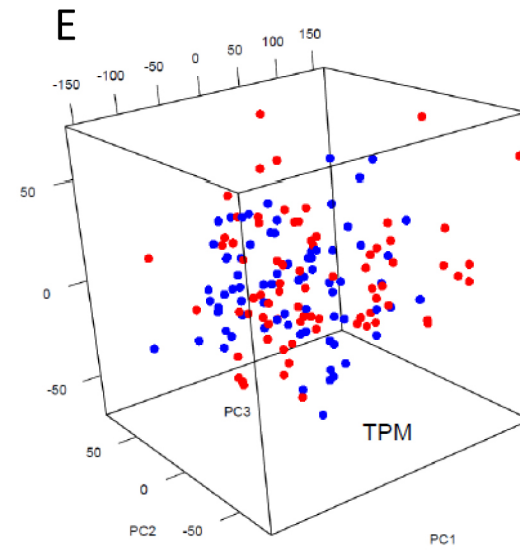

Supplement: Supplementary file 9 — Library size and PCA plots by RIN. A shows the library size (log10) in samples with low RIN values (RIN < 7) or high RIN (> = 9). B-E show PCA plots, colored by samples with low RIN (red) or high RIN (blue), by normalization method. (PDF 2842 kb) [file 12859_2018_2246_MOESM9_ESM.pdf]
